# What do educators need to know about the Torrance Tests of Creative Thinking: A comprehensive review

**DOI:** 10.3389/fpsyg.2022.1000385

**Published:** 2022-10-26

**Authors:** Ahmed M. Abdulla Alabbasi, Sue Hyeon Paek, Daehyun Kim, Bonnie Cramond

**Affiliations:** ^1^Department of Gifted Education, Arabian Gulf University, Manama, Bahrain; ^2^School of Psychological Sciences, University of Northern Colorado, Greeley, CO, United States; ^3^Creativitics, Watkinsville, GA, United States; ^4^College of Education, University of Georgia, Athens, GA, United States

**Keywords:** Torrance Tests of Creative Thinking, school psychologists, teachers, divergent thinking indices, validity and reliability

## Abstract

One of the most important questions that educators try to answer is how to prepare new generations of students for an unpredictable future. Students need to learn several skills, such as creativity, critical thinking, collaboration, and communication (the 4 Cs). Creativity, especially, is an essential skill in a complex and unforeseeable world/era, and an important step in any effort to enhance creativity is to identify students’ creative strengths and relative weaknesses. This review aims to offer school psychologists and other educators such as teachers, policymakers, and curriculum designers a comprehensive and practical guide to one of the most well-known creativity assessments—the Torrance Tests of Creative Thinking (TTCT) that was developed by E. Paul Torrance in the 1960s. The paper discusses the history, components, training, psychometric properties, and uses of the TTCT. Contrary to the notion that the TTCT is only a measure of divergent thinking skills, the current article presents its other uses. It is the authors’ hope that teachers, school psychologists, and other educators will find the information reported in this article useful to better understand the TTCT and use it most effectively.

## Introduction

Education in the 21st century is more complex and challenging than ever before. The United Nations Educational, Scientific, and Cultural Organization (The United Nations Educational, Scientific and Cultural Organization [UNESCO], 2021) lists a number of challenges related to the future of education, such as climate change and creation of educational systems that ensure equity and sustainability. Perhaps the most important question that educators are trying to answer is how to prepare students for the unforeseeable future wherein the emergent problems are still ill-defined (Abdulla Alabbasi et al., 2021). Creativity is a core component in the 21st century skills framework (4 Cs: critical thinking, communication, collaboration, and creativity) (Abdulla and Runco, 2018), but educators are not always sure how to teach and measure creative abilities. This article bridges the gaps in translating research into practice and presents a practical guide to help educators identify and develop students’ creative talents using one of the most well-known assessments, the Torrance Tests of Creative Thinking (TTCT) and to use the theoretical framework underlying the TTCT for teaching creativity in the classroom.

Although previous literature (Treffinger and Isaksen, 2013; Runco, 2016; Abdulla and Runco, 2018) supports that creativity is essential for preparing new generations of learners for an uncertain future, there is a lack of practical guides for teachers to help students identify their creative talents and enhance their creative thinking skills (Abdulla and Cramond, 2017). Despite extensive literature available on the TTCT (e.g., Cramond, 1994; Millar, 2002; Kim, 2006a,b, 2011), this article is unique in that it offers detailed information about the TTCT—from its history of development to the various ways to use it across direct and indirect assessments of creativity. Moreover, this article also covers the components of the verbal and figural forms of the TTCT, as well as information about training, scoring, interpretation of the results, and the uses of the TTCT. The main objective of the current article is to provide educators with essential knowledge and a practical guide about the TTCT and its various uses.

To achieve this objective, this article is divided it into the following sections: (a) the history of the TTCT; (b) the structure and components of the TTCT; (c) the TTCT training, scoring, and interpretation; (d) the validity and the reliability of the TTCT; and (e) the practical implications for school psychologists and other educators.

## The Torrance Tests of Creative Thinking: A historical review

It is interesting that creativity, such an ambiguous and complex topic, was first studied in the 1930s—an era of intelligence research. What motivated Ellis Paul Torrance to study creativity when little attention was paid to it? Indeed, it was his “sensitivity to problems,” which led him to this field of study (For a more complete description of Torrance’s life and work, see Hébert et al., 2002).

The constructs of intelligence and creativity were both initially thought to be singular traits that are largely inherited. Early researchers in the assessment of intelligence, such as Binet, Simon, and Wechsler, used a composite intellectual ability (IQ) score as a measure of intelligence (For a more complete discussion of the development of intelligence testing, see, for example, the book by Flanagan and McDonough, 2018). As research continued, both constructs became broader and more multifaceted. Psychologists such as Spearman, Thurstone, and Cattell developed theories and measures to assess distinct components of intelligence. Then, Guilford (1967) broadened and articulated separate abilities for both intelligence and creativity in his Structure of Intellect Model. Building upon Guilford’s work, Torrance developed his battery of tests called the Torrance Tests of Creative Thinking (originally the Minnesota Tests of Creative Thinking; Torrance and Gowan, 1963).

In the 1930s and 1940s, when Torrance was working as a counselor and a high school teacher in rural Georgia, he struggled with some “difficult” students. Instead of labeling them as “difficult,” Torrance had a positive vision and aimed to understand why some students struggled at school. He concluded that many students were sent to boarding school by their families because of their *off-beat* ideas. Moreover, energetic behavior was not tolerated by their former teachers. Reading the book *Square Pegs in Square Holes* (Broadley, 1943) spurred Torrance to measure creativity. In the book, Broadley (1943) explained that some children are like wild colts who must have their energy directed positively to be useful. From his experience of teaching and counseling students in schools, Broadley’s (1943) description of the wild colts enabled Torrance to see that the “difficult” students have potential (Hébert et al., 2002). Although Torrance believed that all students have creative abilities that can be enhanced, it is more critical to recognize and harness the abilities of those students who may have behavioral and learning issues because of their different way of thinking or those from underrepresented populations whose abilities may not be identified on traditional IQ and achievement tests. Broadley (1943) also described a test developed by Johnson O’Connor titled *Creative Imagination*, which influenced Torrance to develop his own creativity test (as cited in Cramond, 1994), the TTCT, to recognize creativity among his students.

Prior to publishing the official and first version of the TTCT in 1966, Torrance had already published several articles on measuring creativity (Torrance, 1962a,b,c, 1964a,1964b). Torrance initiated the idea of creativity measurement when he was at the University of Minnesota, where he developed the Minnesota Test of Creative Thinking, which was later known as the TTCT (Hébert et al., 2002). The TTCT was also influenced by J. P. Guilford’s extensive work with divergent thinking (DT) (Makel and Plucker, 2008; Plucker et al., 2011).

However, it is important to mention that Torrance’s interest in measuring creativity was not his ultimate goal. Rather, the TTCT was designed as a means to an end, not an end in itself:

I have always been interested in empowering children, releasing their creative potential. But first I had to measure that potential. So I have a reputation as a psychometrician, but all along I have worked with the development of creativity (Torrance, as cited in Cramond, 1994, p. 231).

Torrance had his own vision of the definition of creativity, which reflected the way he measured creativity. Torrance (1966, p. 6) defined creativity as:

A process of becoming sensitive to problems, deficiencies, gaps in knowledge, missing elements, disharmonies, and so on; identifying the difficulty; searching for solutions, making guesses, or formulating hypotheses about the deficiencies; testing and retesting these hypotheses and possibly modifying and retesting them; and finally communicating the results.

What distinguished the TTCT from other creativity or DT tests was not only how Torrance defined creativity, but also how he made it fun, easy to use, and applicable for diverse populations and cultures. Another important distinction from other DT tests was Torrance’s (1979) addition of *creative strengths* to the figural test in 1979. He felt that adding measures for other creative expressions in the list of creative strengths, such as humor, storytelling, and boundary breaking, widened the scope of TTCT beyond DT (B. Cramond, personal communication, 29 November 1980). As the theoretical framework underlying the TTCT covers more than merely DT, the TTCT is valuable in also recognizing and valuing the other creative strengths. As Torrance said:

For any kind of use of the TTCT, it is important that the users have at least basic knowledge of the rationale for the tasks of activities, and be familiar with the concepts of creative thinking that underlie the instrument (Torrance, 2000, p. 1).

## The components of the Torrance Tests of Creative Thinking

The TTCT’s entire battery is composed of Verbal and Figural components and is available in two forms, A and B. Each activity in the TTCT is based on research linking the required ability to creativity (Torrance, 1966, 1974). Table 1 reports the structure of the TTCT Figural and Verbal Batteries.

**TABLE 1:** A comparison between figural and verbal forms of the Torrance Tests of Creative Thinking (TTCT) according to its components, activities, time, changes, and the test nature.

	TTCT figural battery	TTCT verbal battery
Components	1. Fluency (Act. 2 and 3) 2. Originality (Act. 1, 2, and 3) 3. Elaboration (Act. 1, 2, and 3) 4. Abstractness of Titles (Act. 1 and 2) 5. Resistance to Premature Closure (Act. 2) 6. Checklist of Creative Strengths (All Act.)	1. Fluency (All Act.) 2. Flexibility (All Act.) 3. Originality (All Act.)
Activities	1. Picture Construction 2. Picture Completion 3. Lines (in Form A)/Circles (in Form B)	1. Asking 2. Guessing Causes 3. Guessing Consequences 4. Product Improvement 5. Unusual Uses (Cardboard in Form A/Tin Cans in form B) 6. Just Suppose
Time	● 30 min to finish all three activities ● 10 min for each activity	● 45 min to finish all six activities ● 5 min for the first three activities (1–3), and 10 min for activities (4–6)
Changes	Three components added to the TTCT in the 1984 version: Abstractness of Titles, Resistance to Premature Closure, and Checklists of Creative Strengths. The Flexibility has been removed	Unusual Questions activity removed
Test nature	Very little writing required	Relies heavily on writing

It is important to mention that the TTCT Verbal and Figural tests do not measure the same creative abilities. Although two TTCT components are measured in both Figural and Verbal Forms (fluency and originality), the performance on the Verbal and Figural measures shows very little correlation (*r* = 0.06; Cramond, 1994). The little to no relationship was also replicated in a recent study testing 7th graders in Turkey, which showed a weak correlation between figural and verbal originality (*r* = 0.18, *p* = 0.06) and a moderate correlation between figural and verbal fluency (*r* = 0.33, *p* < 0.05; Ulger, 2015). It is crucial that teachers recognize that some students may achieve high scores in one form but not in the other. The two tests differ not just in the format of the responses, verbal vs. figural, but also in the underlying creative thinking components being assessed (see Table 1). Therefore, it would be ideal for teachers to administer both forms to their students to have a better understanding of students’ creative abilities. However, if resources limit testing to one form, it is recommended that the figural be used because schools typically afford students other opportunities to express their creativity in verbal forms. Also, the figural form is more culture fair because of the limited need for writing. A recent study empirically supports that the TTCT Figural test is a more comprehensive and valid measure of creativity than the verbal test examining 994 participants ranging from preschool children to adults (Kim, 2017).

### The Torrance Tests of Creative Thinking figural form

The Figural Form of the TTCT requires responses that are drawn or are pictorial in nature (Torrance, 1972a). With a very little amount of writing required (i.e., for titles), the figural tasks are suitable and appropriate to use in different cultures and socioeconomic levels. In case the examinees are not able to write for any reason, the examiner may assist them (Torrance, 1972a).

In the Figural Form of the TTCT, examinees face three figural tasks: (a) Picture Construction, (b) Picture Completion, and (c) Lines (Form A)/Circles (Form B). Each activity is followed by clear instructions. The Figural Form assesses students’ fluency, originality, abstractness of titles, elaboration, resistance to premature closure abilities, and the Checklists of Creativity Strengths. Flexibility was removed from the Figural test because it correlated very highly with fluency (Hébert et al., 2002).

#### Activity 1: Picture construction

The *Picture Construction Activity* was devised by Torrance to assess the ability of elaborating an idea (Torrance, 1964a). It was also designed to assess the tendency to create meaning out of a seemingly meaningless object (Cramond, 1994). Examinees are required to think of a picture in which the given shape is an integral part (Torrance, 1964a). Examinees have 10 min to finish this activity. Picture Construction activity is scored for originality, abstractness of titles, elaboration, and some items from the Checklist of Creative Strengths. More details will be provided in the scoring and interpretation section.

#### Activity 2: Picture completion (incomplete figures)

The *Picture Completion Activity* (also known as Incomplete Figures) is an adaptation of the Drawing Completion Test, developed by Kate Franck (Torrance, 1964a). It requires the kind of creative thinking that can synthesize and integrate incomplete and relatively unrelated information, in a given structure. The ideas of this activity are adopted from Gestalt psychology, which suggests that an incomplete figure creates tensions to complete it in the simplest and easiest way. Thus, one characteristic of highly creative individuals is the ability to control this tension and delay gratification of this impulse to closure (Torrance, 1964a). This is another creative ability not typically measured by other DT tests. Like the Picture Construction activity, examinees have 10 min to complete 10 incomplete figures. This activity is scored for all TTCT Figural indices.

#### Activity 3: Lines/Circles (repeated figures)

The *Circles/Lines Activity* confronts the subjects with a task that requires different kinds of creative thinking. It confronts respondents with completed forms, which require their completeness to be destroyed to create new forms (Torrance, 1964a). It elicits the tendency to return to the same stimulus repeatedly and perceive it in a new way each time (Cramond, 1994). Examinees have 10 min to complete 30 lines/36 circles. This activity is scored for fluency, originality, and elaboration, in addition to the Checklist of Creative Strengths.

### The Torrance Tests of Creative Thinking verbal form

The Verbal Form of the TTCT consists of six different types of activities: (a) *Asking*, (b) *Guessing Causes*, (c) *Guessing Consequences*, (d) *Product Improvement*, (e) *Unusual Uses*, and (f) *Just Suppose*. The *Unusual Questions* activity was removed from the verbal battery because it added length to the battery without adding significant information. In the Verbal Form of the TTCT, examinees are provided with a picture (stimuli) in each activity with verbal tasks, which they respond to in writing. The TTCT Verbal Battery assesses students’ fluency, flexibility, and originality abilities. Examinees have 45 min to complete all six verbal activities.

The first three activities: Asking, Guessing Causes, and Guessing Consequences or (Ask-and-Guess), are based on research linking curiosity to creativity. Activity 4, Product Improvement, is linked with a kind of creative productivity called “improve existing product,” which is different from the kind of creative productivity wherein the individual has to create something that does not exist. The Unusual Uses activity encourages examinees to think of alternative uses for a common object such as wheels. The last activity, Just Suppose, is a hypothesis about an improbable situation, which assesses individuals’ tolerance to and playfulness with unusual situations (Cramond, 1994).

## The Torrance Tests of Creative Thinking administration, scoring, and interpretation

Because one does not need special training to administer the TTCT, teachers can do so with the aid of the manual. Although as with all standardized tests, it is important to follow the directions and timing carefully, the instructions are clear [For a recent discussion on time-on-task and instructions in DT tests, see Said-Metwaly et al. (2020) and Paek et al. (2021)]. Because the figural and verbal forms of the test can each be administered within 1 hour, they are designed to fit within the school day.

Special training is needed to learn how to score the TTCT reliably. Understanding how to score the TTCT is important because the TTCT is used in some states to identify children for gifted programs (Kim, 2009), as creativity is taken as an essential criterion in many giftedness definitions (Renzulli, 2005). Besides broadening the thinking skills measured to better match the definition of giftedness, it allows schools to identify gifted children from under-represented groups who may not be identified based on standard IQ and achievement tests. Furthermore, it helps educators understand what being good at creative thinking specifically means so that they can apply this meaning to their own creativity pedagogy in their practice. As Torrance was originally motivated by the behavior of some students seen as “difficult,” finding creative strengths of some students may also help educators in designing individual learning programs for these students. Many highly creative children may be referred for testing because of behavioral issues or learning disabilities (Torrance, 1961). Scoring the TTCT and interpreting the scores will allow school psychologists to advise teachers about ways to promote creative thinking in measurable ways. School systems that do not have trained scorers can send the completed tests to Scholastic Testing Service, the publisher of the tests, for scoring.^1^

### Training for scoring

To ensure qualified scorers for the TTCT, ongoing training is offered by the Torrance Center for Creativity and Talent Development at the University of Georgia for both Verbal and Figural Forms. Trained school psychologists, who already are familiar with testing procedures and scoring should not have difficulty learning to score the TTCT. Studies of scorer reliability indicate that individuals specially trained and experienced in scoring the TTCT are capable of scoring them with a very high degree of reliability (Torrance, 2008b). Despite the documented evidence of scorer reliability on the TTCT, it is still important for scorers to accurately and comprehensively understand the underlying purpose and mechanism of assessment in the TTCT and specific protocol to follow in scoring. Otherwise, a substantial amount of variability is likely in scores yielded by scorers with different backgrounds and understanding. Yarbrough (2016) examined variability at the item-level in 35 Turkish scorers and suggested that ambiguity of test takers’ responses, cultural and linguistic backgrounds of scorers, and the lack of scorers’ clear understanding about individual indices can threaten scorer reliability, which highlights the importance of training for scoring.

### Scoring

The latest version of the TTCT’s Figural and Verbal Batteries (Torrance, 2017) consists of the following indices:

#### Fluency

**FIGURE 1:**
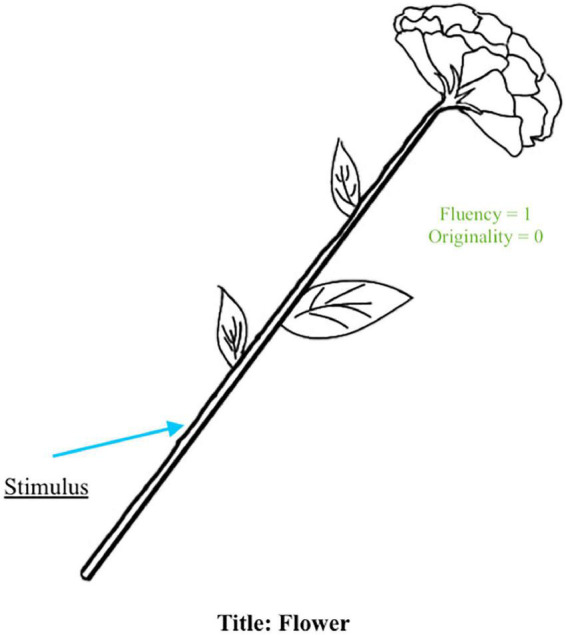
Example of a response where the stimulus is identifiable and used as part of the idea (i.e., flower).

*It is the number of relevant responses*. It is scored in both Figural and Verbal Batteries. In scoring Figural fluency, two important criteria must be met; the stimulus must be *identifiable* and *used*. However, scorers should be careful in scoring the first criterion because some examinees’ drawings may not be easy to identify. In such a case, it is always good to ask another scorer if they could identify the picture drawn by the examinee or ask the examinee what they drew. In scoring Verbal fluency, the scorers should be sure that the examinees’ verbal responses are relevant to the tasks given. Fluency in both Figural and Verbal Batteries is the “gatekeeper” for scoring the other TTCT’s components; in other words, any responses that do not meet the criteria for fluency are not scored for anything else. Figure 1 shows an example wherein the stimulus was used as part of the drawing, and thus, the student received one point in fluency for their response.

#### Flexibility

*It is the number of different categories or shifts of ideas*, which is scored only in the Verbal Battery. Scorers categorize responses using the list of categories provided in the Verbal Manual for Scoring and Interpreting Results. Examinees gain one point for each new or different category.

#### Originality

**FIGURE 2:**
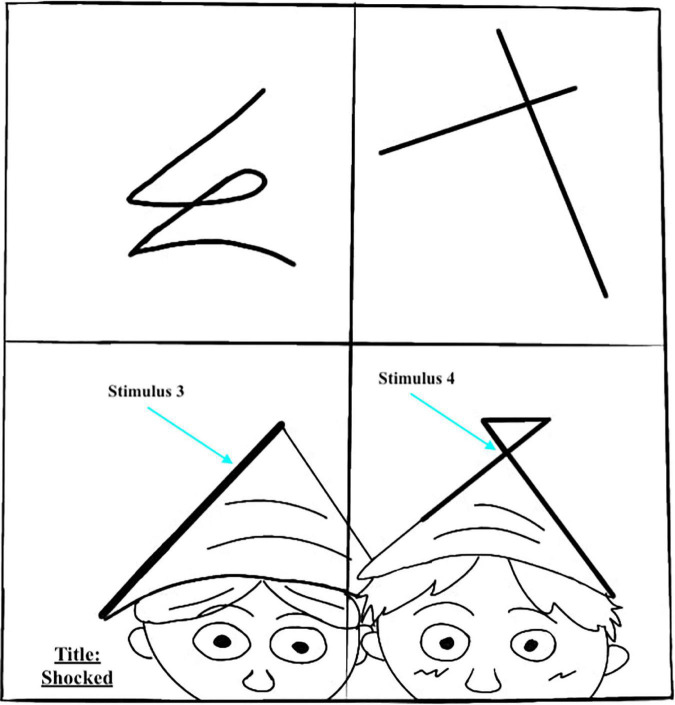
Example of combining two incomplete figures.

*It is the number of unusual ideas as determined by statistical infrequency*, implying that rare ideas are original. The scoring guide includes a list of common responses. If the response is not on the list, it is given a point for originality. In Figure 1, the idea of a flower is common for this stimulus, and therefore, the student received a score of zero in originality. Examinees could gain additional points (bonus) in the Figural Form in specific cases (i.e., combining two or more incomplete figures; see Figure 2), which are illustrated in the test and training manual. The list of common responses may change with the passage of time and from one culture to another; therefore, the TTCT is normed separately in different cultures and re-normed every 10 years to account for differences by cultures and generations.

#### Elaboration

**FIGURE 3:**
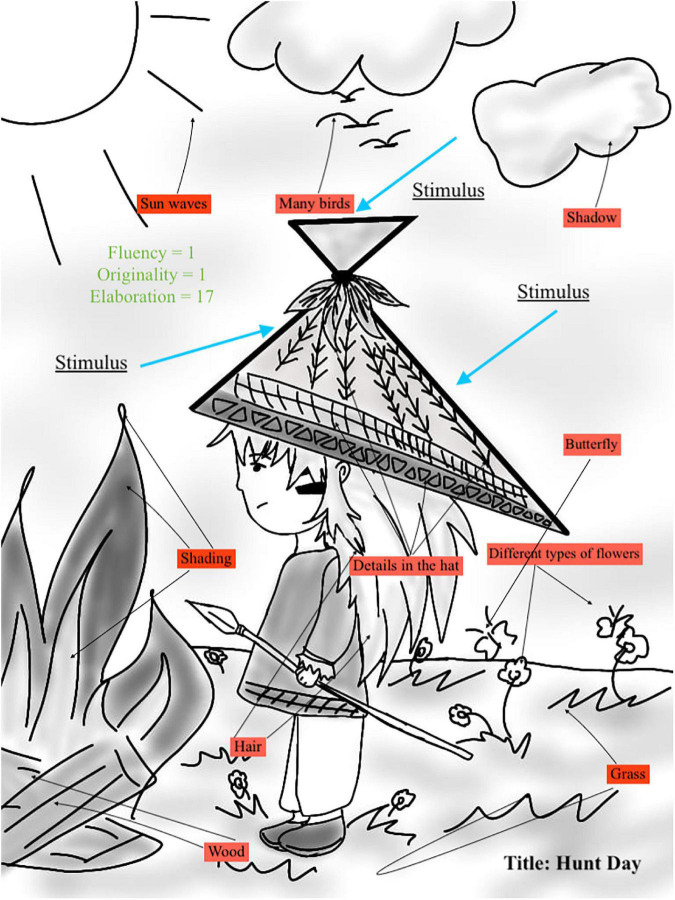
Example of scoring elaboration.

*It is the addition of ideas beyond the minimum necessary for the response*. To determine what elaboration is and is not, it is always good to think of the basics. For example, the basics of a door are the door and the knob. Any additional elements such as the door design or dimensions are considered as elaborated detail. As people may disagree on what the basics are, there is a range for each point as shown in the Streamlined Scoring Sheet. For instance, examinees receive 1 point if the additional ideas range between 0 and 5, and 2 points if the additional ideas are between 6 and 12 ideas. Figure 3 shows an example of elaboration.

#### Resistance to premature closure

It is a gestalt measure of a person’s ability to stay open and tolerate ambiguity long enough to come up with a creative response. In his article entitled “Resistance to Premature Closure as a Possible Indicator of Incubation Ability,” Torrance (1979) stated:

Evidence from many sources makes it clear that once the human mind attains closure on a problem, i.e., jumps to a conclusion, the incubation of original, breakthrough ideas is unlikely. Ability to maintain openness is influenced by motivation and the use of certain deliberate creative problem-solving procedures (p. 59).

Resistance to premature closure is scored only in Activity 2 of the Figural Form. Each incomplete figure has one or more focus areas that create tension to close them.

#### The checklist of creative strengths

**FIGURE 4:**
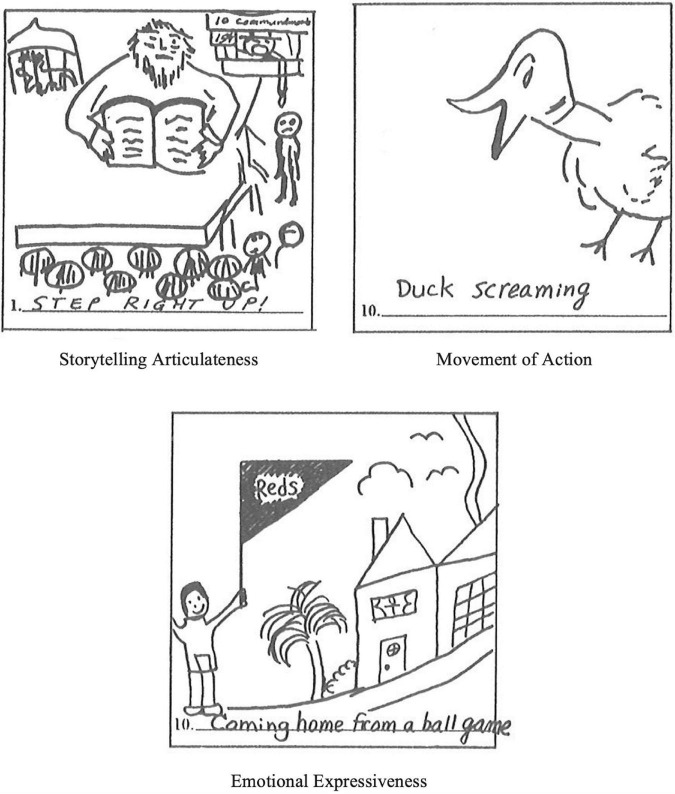
Example of responses on three creative strengths indices.

Thirteen criterion-referenced measures were added to the TTCT Figural Battery in 1984 (Torrance, 1990). These include Emotional Expressiveness, Storytelling Articulateness, Movement or Action, Expressiveness of Titles, Synthesis of Incomplete Figures, Synthesis of Lines or Circles, Unusual Visualization, Internal Visualization, Extending or Breaking Boundaries, Humor, Richness of Imagery, Colorfulness of Imagery, and Fantasy. Figure 4 shows examples of three Creative Strengths.

### Interpretation

The TTCT only assesses creative *potential*, more specifically, the creativity *process* among many elements of creativity. Torrance himself believed that administering the full battery of Verbal and Figural Forms to an examinee would not guarantee that they would behave in a highly creative manner; creative productivity also requires other factors, such as motivation, skill, and opportunity (Cramond, 1994).

After the scoring process is conducted by a well-trained school psychologist, the scores need to be interpreted in a meaningful way using the Figural and Verbal Norms-Technical Manuals. For each grade level and age group, there is a table that converts the raw scores of each component and the composite measures-average to the national percentile. The tables start from (00) for pre-school children and cover all grades from 1 to 12 and adults. The age norms range from 5 to 20 years old. The age-related derived measures are identical to those given for grade-related groups (Torrance, 2008a). There are six categories for interpreting the average standard scores: (a) *Weak* (0–16%), (b) *Below Average* (17–40%), (c) *Average* (41–60%), (d) *Above Average* (61–84%), (e) *Strong* (85–96%), and (f) *Very Strong* (97–100%). Although the overall score indicates examinees’ standing in their creative potential on average, we strongly recommend looking at each sub-score in addition to the average scores because the sub-scores are fairly informative about the different aspects of creative strength: some students, for instance, may score low or average overall, but high on some subtest(s) indicating that some of their creative strengths could be uncovered otherwise.

## Psychometric qualities of the Torrance Tests of Creative Thinking

### Reliability of the Torrance Tests of Creative Thinking

Because there is no universal agreement on what creativity is, except that it requires both originality and appropriateness (Runco and Jaeger, 2012), or on how to measure it, many creativity assessments are available. The same is true of intelligence. Teachers know that their students have many different backgrounds, abilities, and potentials. Their students’ creative potential profiles should be as diverse as they are. For example, Tony may prefer drawing, whereas Nataly may prefer reading. Garrett is hyperactive, but Jean is introverted. Some teachers might be interested in identifying the creative potential in their bright, ESL (English as second language) students who cannot speak English well; others may implement a culturally responsive curriculum to support African American students; and some may want/need to support students from low SES families. It may be very challenging for teachers to do many kinds of assessments. Although students from diverse backgrounds may take various kinds of assessments, educators may be dubious about the results, and may raise the following questions: “Can this assessment measure their creative potential accurately (or consistently) across diverse populations?” or “Can this assessment really predict their creative achievement?” The former is labeled as reliability and the latter as validity. Reliability and validity are crucial components for valid or trustworthy psychological measures. The TTCT has proved high reliability and validity for the last six decades, through application and validation in over 2,000 studies worldwide, in 35 languages (Millar, 2002) such as Arabic (Said-Metwaly et al., 2021), Persian (Karimi et al., 2010), Kiswahili (Humble et al., 2018), Turkish (Bart et al., 2017), Spanish (Krumm et al., 2016a,b), Korean (Yoon, 2017), and Japanese (Higuchi et al., 2012). Therefore, the TTCT can measure the creative potential consistently across diverse backgrounds with minimal cultural, language, and IQ bias (Torrance, 1966, 1974). Moreover, it can predict creative achievements concurrently and for the future (Runco et al., 2010; Erwin et al., 2022).

There are three activities in the Figural test and six activities in the Verbal test. Items within each activity were established to assess the same construct—creative potential—consistently across items. Reliability indicates how each activity measures the same construct consistently (Barlow and Proschan, 1974). High reliability implies that students who score high on one activity should also score high on the other activity. In 2016, the Scholastic Testing Service, Inc., (STS) investigated the reliability of the tests with nationwide samples, which ranged between 0.87 and 0.97 (Torrance, 2017), indicating good reliability. Some people may raise questions about whether different raters could make different decisions on scoring the same test. The agreement among raters was 90% and above in the 2016 STS study with 60,917 samples nationwide, including students from kindergarten through 12th grade (Torrance, 2017). This indicates that the TTCT is solid in terms of consistency.

### Validity of the Torrance Tests of Creative Thinking

#### The Torrance Tests of Creative Thinking on predicting creative achievements concurrently

A measure consists of items that are supposed to investigate the same abstract attribute or quality operationally defined with a theoretical basis. Therefore, it is important to ensure that all items measure the same construct; this is called construct validity. Construct validity indicates how well items represent the construct being examined (Cronbach and Meehl, 1955). The TTCT was designed to measure six sub-constructs of creativity and creative strengths: fluency, flexibility, originality, elaboration, titles, closure, and creative strength. There are a few studies wherein the TTCT measured one dimension of creativity (Hocevar, 1979) and sub-indices of the TTCT were moderately correlated (Runco et al., 2010). However, a composite score of all indices was not suggested, as it was felt that a single score could not represent all different aspects of creative potentials (Torrance, 1966). However, the composite score could be considered as overall creative energy (Torrance, 1995). Recent research has suggested that the TTCT consists of at least two different factors—innovative and an adaptive orientation (Kim, 2006b). Sub-indices and the composite score were used to predict many types of creative behaviors for diverse developmental groups.

#### The Torrance Tests of Creative Thinking on predicting creative achievements in longitudinal studies

Torrance was interested to see the possibility of how the test would influence later creative productivity. The development of creative assessment fundamentally aims to measure creative potentials that translate into creative achievements in the real world, at present or in the future. How long can a test reliably predict futuristic creative achievements? How about 1 year, 10 years, or even 50 years? Obstacles in conducting longitudinal studies are expected: deficit of funding; mobility of students, which is daunting because it is difficult to track participants over years; disparity of pencil and paper tests from the real world; accomplishments; unexpected factors in longitudinal studies; and a 50-year gap from 1958 to 2008.

##### The four follow-ups of elementary school students at 22-, 40-, and 50-year intervals

The most recently completed longitudinal study (Runco et al., 2010) showed that the TTCT still holds moderate correlations with later creative achievements in personal domains over 50 years later, but not in public domains. This longitudinal study was initiated in 1958 to show the long-term predictability of the TTCT. There were four follow-ups for two different initial groups. It would be more reasonable to show everyday creativity, rather than publicly recognized creativity for people in their 60s, as specific criteria are more fitting for those specific samples (Runco et al., 2010). Runco et al. (2010) found an optimal level of DT in their recent longitudinal study. In other words, too much divergence in thinking may not work for most achievements, whereas DT could contribute to creative achievements up to the optimal degree.

The initial study was conducted with elementary school students enrolled in grades 3–5 at two Minneapolis elementary schools (Torrance, 1981). Students completed various instruments: TTCT, a biographical inventory, creative writing samples, checklist of creative activities performed on their own, an intelligence measure, and socio-metric questionnaires. The first follow-up was in 1979–1980, after 22 years, when 211 boys and girls turned 30–32 years old. In the study, five criteria for creative achievements were rated by expert judges: (a) number of high school creative achievements, (b) number of post high school creative achievements, (c) number of “creative style of life” achievements, (d) quality of highest creative achievements, and (e) creativeness of future career image. All correlation coefficients between the TTCT and the five criteria of creative achievements ranged from 0.38 to 0.58 (Torrance, 1981), which is conventionally interpreted as moderate to large effects (Cohen, 1988). Forty years after the initial testing, the predictability of the TTCT was conducted again with the same predictors and criteria for creative achievements (Cramond et al., 2005). It included 99 respondents—one-fourth of the initial sample. The predictive validity of Fluency, Originality, and IQ in childhood was significant and ranged from 0.23 to 0.30, which could be interpreted as a moderate effect (Cohen, 1988). In contrast, IQ, Flexibility, Originality, and the creative index significantly predicted the quality of creative achievements. In the study, the TTCT contributed to 23% of the variance in creative productivity, which is impressive, considering the span of 40 years. This finding implied other important mediators, such as motivation and opportunity to bloom creative potential.

##### The two follow-ups of high school students at 7- and 12-year intervals

The 1959 study of Minnesota high school students included 69 participants. Of them, 67% completed measures after 7 years, initiated in 1966. In this study, intelligence, high school achievements, and peer nomination of creativity were used as predictors along with the TTCT scores (Torrance, 1969). The criteria for creative achievements included three indices: (a) quantity of creative achievements, (b) quality of creative achievements, and (c) creative motivation (Torrance, 1972b). All fluency, flexibility, and originality showed significant predictive validity coefficients ranging from 0.34 to 0.48, larger than intelligence, high school achievement, or peer nominations that ranged from 0.09 to 0.37 (Torrance, 1972b). This implied that the TTCT scores predict later creative achievements better than intelligence, high school achievement, or peer nominations. The second follow-up was conducted 5 years later in 1971. There was high correlation between the sets of predictors and criteria (0.59).

## Practical implications

### Identification

In the current article, we suggest a few ways of using the TTCT. Of course, the TTCT has been used as a formal assessment for gifted identification. E. Paul Torrance developed the TTCT to assess creative potential, and discover, release, and nurture children’s creativity (Torrance, 1966, 1974, 1979). Creativity is one of the criteria for being identified as gifted in 31 states out of 44 states in the USA participating the nationwide survey conducted by the National Association of Gifted Children (Rinn et al., 2020) and is becoming increasingly important to other countries (Abdulla Alabbasi, 2020; Ayoub et al., 2022). For example, because European countries have recognized the importance of creativity, the Programme for International Student Assessment (PISA) chose Creative Thinking as the innovative domain for the 2021 testing and enlisted the aid of the LEGO Foundation to investigate possible assessments (McClure and Jaeger, 2020). Asian countries have also seen an increase in interest in the nurturance of creativity in students (Alnabhan et al., 2014; Cramond et al., 2020). The TTCT has been used to measure creativity, and the TTCT composite scores have been used to identify students for gifted programs like other standardized scores, such as achievement tests (i.e., reading or math tests) or mental ability tests (e.g., CogAT). In addition, the TTCT detailed scores (fluency, originality, elaboration, abstractness of titles, resistance to premature closure, and 13 Creative Strengths) are helpful in profiling individuals’ creative strengths and weaknesses. This profiling can provide additional information and individualized instruction for each person in their creative development.

In the overall gifted identification process, the conceptual basis of the TTCT can also be employed for informal assessment when teacher nomination or referral takes place. If teachers understand the theoretical underpinning of the TTCT, they would be better aware of the ways in which creative students express their creativity. For instance, the scoring indices of the TTCT, such as fluency, originality, elaboration, flexibility, resistance to premature closure, humor, and storytelling, may also serve as behavioral indicators when teachers observe students in the classroom.

At the same time, informal assessments, including homework, writing samples, discussions, projects, and art products, are undertaken through daily learning activities in the classroom (Navarrete et al., 1990). Such informal assessments are critical in providing students with educational opportunities that meet their needs, as these assessments often compensate for formal assessments.

Schools implement screening procedures based on formal assessments to find students with potential giftedness, who then complete more formal identification assessments. However, there may exist creative students who are not recognized through formal assessments owing to several reasons, such as the lack of motivation or academic excellence and behavioral issues. Thus, nomination takes place based on informal assessments to compensate for screening, and teachers or parents are commonly involved in the identification process thereof. Such assessments are widely used as an entry point, especially for elementary school students in the gifted program identification process (McBee et al., 2016; Callahan et al., 2017). Teachers generally observe and evaluate students in the classroom and nominate them to be tested for a gifted program based on a standardized checklist of gifted behaviors (a formal teacher-rating instrument) and high achievement test scores. As the nomination stage is part of the screening for gifted program identification, many gifted students are missed at this stage and never get tested.

McBee et al. (2016) investigated the effect of the nomination stage on gifted program identification and found that the reliability of nomination was poor. Teachers might have different implicit theories of giftedness based on their different backgrounds and experiences, heavily influencing the nomination process. Therefore, all possible gifted students from diverse cultural backgrounds, who might not align with the teacher’s traditional expectations for gifted students, could miss out on being nominated for gifted program referrals. For example, gifted Black males tended to be under-represented in gifted programs compared to other populations because their giftedness, including creativity, was not easily identified through the traditional lens of giftedness.

Torrance contributed to redefining giftedness and different types of intelligence (Grantham, 2013). As a result, underrepresented populations (ethnic minorities, low-income students, and English as second language learners) benefited from the multiple criteria for giftedness, and their creative strengths became recognized. Thus, if teachers and educational practitioners are aware of the theoretical frameworks of the TTCT, including creative strengths, and use them as a tool for informal assessment in a classroom, they will have more opportunities to recognize and nurture students’ hidden creative potentials.

In addition to identifying students for a gifted program, the TTCT can be used to find strengths in students who do not perform well in traditional classrooms. Such students may be seen as having behavioral problems or may have learning disabilities that prevent them from high performance in highly structured academic settings. Yet, these students might flourish in a classroom with activities designed to develop their creative strengths. For example, Steven Jobs was becoming quite a discipline concern until he got a teacher who recognized his creativity and got him started on building things. Jobs credited that experience, along with opportunities to build with his father, for the inception of his fascination for building things (Isaacson, 2011).

### Teaching in the classroom

Teachers can also use the TTCT framework to guide their pedagogy in supporting student creativity in the classroom. They can integrate the TTCT framework into their instructions and warm-up activities promoting students to think creatively. Warm-up activities have been tested to encourage students to be cognitively flexible before they take on creative tasks (Aliotti, 1973; Nash, 1975; Dunphy and Milbourne, 2009). To develop creativity in the classroom, one of the most critical factors is a proper learning environment, wherein students do not feel their thoughts and behaviors to be judged or evaluated (Beghetto and Kaufman, 2014). Activities similar to those of the TTCT can be used as warm-up activities that can contribute toward a psychologically safe classroom environment.

We suggest using various strategies such as multimedia, multisensory approaches, a sense of humor, or movement to encourage students to generate many new and detailed ideas, while doing warm-up activities based on the TTCT. For example, there are open-ended questions to guide students to answer with multiple solutions. Torrance (1979) (as cited in Cramond et al., 2005) developed the Demonstrator Form, which is a short version of the TTCT consisting of two figural activities and two verbal activities. One of the verbal activities is about product improvement: “Try to improve this stuffed toy rabbit so that it will be more fun to play with.” Students are encouraged to try and think of as many ideas as possible (Fluency), things that no one else will think of (Originality), and additional details to make their ideas more elaborate (Elaboration). This warm-up exercise provides not only a psychologically safe classroom environment, but also an opportunity for students to take initiatives of thinking and speaking up about their thoughts which are essential in effective learning. More sample warm-ups are described in Table 2.

**TABLE 2:** Torrance Tests of Creative Thinking (TTCT) components and corresponding warm-up examples.

Warm-ups	Description	TTCT components
Brainstorming	Encourage students to generate many ideas to find a solution to the problem. Usually, this is used in groups as a problem-solving exercise in spontaneous discussion settings.	Fluency, flexibility
Encounter lesson	Imagine being something. It could be anything from any subject area (i.e., historical events, characters in books). This helps students to learn to emphasize or better understand by taking others’ perspectives.	Fluency, originality, flexibility
Force fit	Ask students to combine two or more seemingly unrelated concepts from different subject areas.	Originality, flexibility, resistance to closure
Guided visualization	Read a story and ask related questions. This helps students to understand and build up their own images related to the story.	Imagination, visualization
Inkblot	Blot ink or paint on a piece of paper and fold the paper in half horizontally and vertically (there will be four sides). Each student looks at one side of the inkblot and writes down what he/she sees. Then, the students share their perspective.	Fluency, originality, elaboration, flexibility, unusual visualization, emotion
SCAMPER (Eberle, 1996)	SCAMPER means Substitute, Combine, Adjust, Modify, Put to other uses, Eliminate, and Reverse. One or all of these thinking methods can be chosen to solve the problem.	Elaboration, flexibility
Six thinking hats (De Bono, 2004)	Each hat’s color represents a different view- White (neutral objectivity), Red (the emotional view), Yellow (the logical positive), Green (creativity), Blue (process control), and Black (the logical negative). Students can look at a problem from six different perspectives with this thinking process.	Flexibility
Spinning a yarn	Tie knots with different lengths and make a ball of yarn. Begin by telling an imaginary story until a student get to a knot. Then, the next student takes the string, and continues to tell imaginary story until they get to another knot.	Fluency, elaboration, resistance to closure

Further, the TTCT frameworks can be used as effective instructional strategies. In any subject class, the TTCT-Verbal questions (Asking, Guessing Causes, Guessing Consequences, Product Improvement, Unusual Uses, and Suppose) and the creativity criteria (Fluency, Originality, Elaboration, and Flexibility) can be used as tools to complement lesson plans and course objectives. For instance, a teacher teaching about the American Civil War in a Social Studies class. In this class, the learning objectives would involve understanding the historical and cultural background of the American Civil War, comparing and contrasting the North and the South (economic situation, government, political characteristics, lifestyle, etc.), and learning about the outcome of the American Civil War. Although the learning objectives may vary slightly by the grade level, adapting the TTCT components to the class, the teacher could, for instance, give a writing assignment to students with the prompt “What if there was no American Civil War?” Students would need to generate as many ideas as possible (Fluency), original ideas others cannot think of (Originality), and additional details based on the content they have learned from class about the American Civil War (Elaboration). In addition, several creativity strategies, such as cause-consequences, compare-contrast, and perspective-taking, can be applied to achieve the learning objectives. This example is just one of the many instructional strategies that teachers can develop using the TTCT frameworks in any subject area including a writing class that empirically tested the effectiveness of the TTCT related strategies in Jordanian students (e.g., Rababah, 2018).

## Conclusion

The main objective of the current work was to offer a comprehensive and a practical guide for school psychologists and teachers who want to know about and use the TTCT in their instructions. We tried to summarize information about the TTCT from the tests’ manuals, Torrance’s original works, what other researchers wrote about the TTCT, and from the authors’ personal experience with the TTCT. Of course, the TTCT is not the only measure of DT and we do not argue that it is the best assessment of creative thinking. However, as discussed in the Historical Review, various factors make the TTCT one of the best choices for school psychologists and teachers who are interested in identifying and nurturing the creative potential of students. Cumulative evidence on the validity and the reliability of the TTCT in different cultures supports such an argument. Finally, contrary to the notion that the TTCT is only a measure of creative thinking, this article discusses the different ways in which the TTCT can be used. We hope that school psychologists, teachers, and other readers who are interested knowing about the TTCT, will find this article useful.

## Author contributions

AA conceived the idea of this work and wrote the introduction, the historical review of the TTCT, and the components of the TTCT. SP wrote the TTCT training, scoring, and interpretation section as well as psychometric qualities of the TTCT. DK wrote the practical implications of the TTCT. BC revised the manuscript and added the conclusion. All authors contributed to the article and approved the submitted version.

## Footnotes

1
https://www.ststesting.com/gift/index.html
